# Medical student attitudes on vaccination relevance: A mixed-method study

**DOI:** 10.1371/journal.pone.0273529

**Published:** 2022-08-24

**Authors:** Anne Herrmann-Werner, Teresa Festl-Wietek, Christian Gille, Stephan Zipfel, Steffen Wiechers

**Affiliations:** 1 Tübingen Institute for Medical Education, University of Tuebingen, Tuebingen, Germany; 2 Department of Psychosomatic Medicine and Psychotherapy, Internal Medicine, University Hospital Tuebingen, Tuebingen, Germany; 3 Department of Neonatology, Pediatrics, University Hospital Heidelberg, Heidelberg, Germany; 4 Private Practice for Pediatrics, Adolescent Medicine, Pediatric Pulmonology, Tuebingen, Germany; University of Mississippi, UNITED STATES

## Abstract

**Background and objectives:**

The study aims to investigate the attitudes of medical students regarding the importance and relevance of vaccinations, whether vaccinations should be compulsory and how to employ a new teaching concept to deal with vaccination-critical parents.

**Methods:**

This mixed-method study consists of a quantitative questionnaire and focus groups. Quantitative data were analysed by calculating the descriptive statistics, and interviews were analysed using Mayring’s content analysis.

**Results:**

A total of 170 medical students completed the questionnaire, and 59 students participated in 9 focus groups. Students reported that they felt more confident dealing with vaccination-critical parents after learning the new teaching concept. Similar results were found for medical students prior to and during the pandemic. During the pandemic, medical students viewed vaccinations for several diseases, such as measles or COVID-19, as important (range: *M* = 3.56, *SD* = 0.54 to *M* = 3.97, *SD* = 0.17). Similar results were found for medical students prior to the pandemic (range: *M* = 3.26, *SD* = 0.77 to *M* = 3.94, *SD* = 0.24). In the focus groups, however, medical students displayed controversial attitudes regarding compulsory vaccinations.

**Conclusions:**

While the medical students agreed on the use of vaccination for highly infectious diseases, their level of agreement decreased depending on the severity of the disease. Practical recommendations that come out of the study are creating a trustful relationship with and delivering information to patients.

## Introduction

The timely application of vaccinations against preventable diseases is of particular importance in paediatrics. The vast majority of the population accepts the necessity of preventive medical check-ups for children and adolescents [1]. Regular check-ups offer an opportunity for individual health counselling and the detection of behaviour that is detrimental to health or contributes to the development of diseases and risk factors (e.g., obesity, unhealthy diet, limited social behaviour and lack of exercise) [1]. Vaccinations are included in these check-ups in accordance with the scheme promoted worldwide by the World Health Organisation (WHO) [2] and by the Standing Committee on Vaccination (Ständige Impfkommision; STIKO) in Germany [3], thereby making vaccination the standard of care from early childhood on. Regular childhood medical check-ups are an especially important venue for vaccination, particularly because adults often do not attend regular medical check-ups, impeding the success of later-in-life vaccination campaigns.

The COVID-19 pandemic resulted in a significant rise in awareness of the role of vaccination. Initially, social distancing, quarantine and hygienic measurements were the only responses to COVID-19 [4]. However, the hope of truly fighting the virus was only aroused with the availability of COVID-19 vaccinations in late 2020. Interestingly, while hope was raised by the development of successful vaccinations, this was directly paralleled by vaccination scepticism [5]. In general, vaccinations are implemented to prevent the outbreak of dangerous, infectious, and sometimes incurable diseases, which makes them an essential preventive measure in the current medical practices.

Preventive visits including vaccinations decreased during the COVID-19 pandemic, and public health advocates warned that this drop could continue for vaccination-preventable diseases, because parents would not see these vaccinations as relevant [6]. Based on data from recent outbreaks (e.g., measles in the U.S. in 2019), unvaccinated or unimmunised children can miss important steps in their educational development, because they are excluded from everyday activities and required to stay out of school [7]. In a survey of attitudes toward COVID-19 vaccinations, Paul et al. (2021)asserted that general mistrust in vaccinations and concerns about side effects were relevant reasons to decide against vaccinations [8]. Talarek et al. (2021) reported, however, that attitudes toward vaccinations might change during an outbreak [9]; to change the attitudes related to vaccinations, physicians should deliver understandable medical information to the patients [7, 10].

Even though this demonstrates why the importance of vaccination-related attitudes in relation to the enormous potential of vaccination should be an integral component of medical education and it also is common practice in medical schools, this aspect has received little attention in undergraduate medical curricula worldwide and the transfer of knowledge regarding the contents or preventive factors of vaccinations has been ineffective thus far, despite up-to-date, evidence-based, readily available information [11]. Moreover, the course program on this topic should be enhanced.

In Germany, STIKO developed an app that quickly offers access to information, but despite such easily accessible information, communicating this information is often described as the most difficult part of clinical practice [11–13].

As of 2019, the WHO declared that vaccination scepticism was one of the top ten global health threats [14]. Moss et al. (2016) investigated patient–physician communication regarding adolescent vaccination and reported that vaccination rates were higher when parents were given understandable information and practical recommendations about vaccinations by their physician [15]. In several studies Opel et al. assessed the influence of providers’ communication and behaviour on parental vaccination acceptance and visit experience and found that parents prefer to accept the vaccinations when their physician initiated presumptive formats in the discussion, and even when parents demonstrated resistance in the discussion, they tended to accept the vaccination if their physician offered vaccination recommendations [16, 17].

In particular, dealing with vaccination-critical parents presents a constant obstacle in the area of patient contact [13, 18, 19]. Medical professionals—especially those with little experience and starting their careers—are faced with the challenge of effectively communicating appropriate information about necessary vaccinations to parents; this can be avoided to some extent by appropriate preparation [18]. As the numbers of vaccination-critical parents are increasing, this poses a significant problem for health policy [20, 21].

In this way, patient–physician communication plays an essential role in vaccination acceptance and should be integrated into medical training [22]. Thus, physician involvement in vaccine uptake is important and it would be good to train medical students how to deal with vaccine critical parents. In order to increase medical students’ involvement in vaccine uptake we need to know their attitudes towards vaccines.

### Aim

This study aimed to investigate the attitudes of medical students regarding the importance and relevance of vaccinations and vaccination-critical parents and their arguments for and against vaccinations in general and compulsory vaccinations in particular. Prior to and during the pandemic, medical students learned a newly developed teaching concept on (1) how to receive evidence-based information through modern knowledge media, (2) how to convey this information in parent discussions about vaccinations and (3) how to respond to typical counterarguments.

## Methods

### Design, participants and procedure

This study followed a mixed-method design that included a quantitative questionnaire and focus groups on medical students’ attitudes toward vaccinations. The study was conducted at the University Hospital of Tuebingen in the summer of 2019 and the 2021– 2022 winter. A teaching concept on prevention in paediatrics was newly developed and medical students were invited to participate in the study; all students were in their fifth year of study. One portion of the course focused on the topic of vaccinations; it started with a 20-minute theoretical introduction, followed by a simulated patient–physician encounter wherein one of the students conducted a conversation with a vaccination-critical parent on the MMR (i.e., mumps, measles and rubella) vaccination. After attending the course, students were expected to know how to deal with vaccination-critical parents, specifically how to understand parents’ fear of vaccinations and deliver understandable information to them representing the outcomes of the course. The course was developed by two experts (one expert in medical didactics and one expert in paediatrics). The medical students were randomised into two groups. Group 1 of medical students completed a quantitative questionnaire regarding their attitudes on vaccination before learning the new teaching initiative, and group 2 participated in focus-group discussions on vaccinations after the simulated patient–physician encounter to clarify their opinions on vaccinations. The responses of the students in group 1 were given separately to avoid biasing students (Group 2) in the focus groups. Altogether, the length of the course was 90 minutes; while the quantitative questionnaire was distributed among medical students prior to and during the pandemic, the medical student focus groups were only conducted before the pandemic.

### Ethics

This study was approved by the Ethics Committee of Tuebingen Medical Faculty (493 / 2018BO2). All participants provided written informed consent and participated on a voluntary basis.

### Questionnaire

The questionnaire was a self-reporting instrument that included items on the importance and relevance of vaccinations in general. One example question on the importance of vaccinations was, ‘How important do you consider a vaccination for measles?’ All items were rated on a 4-point Likert scale from 1 (not at all) to 4 (very). Students could also rate reasons for and against vaccinations. One example reason in favour of vaccinations was, ‘Vaccination saves lives’; and an example reason against vaccination was, ‘The potentially preventive disease is not dangerous.’ The medical students rated on a 5-point Likert scale from 0 (not at all) to 4 (very) their level of agreement with several statements, such as ‘Vaccinations are important to a child’s health’, and they could choose reasons for or against vaccination by indicating ‘Yes’ or ‘No’. Finally, demographics, such as age and gender, were collected.

The questionnaire distributed in the winter semester 2021/2022 was supplemented with questions on COVID-19 related to the importance or relevance of the vaccinations. The questions on Covid-19 were the same open questions rated on a 4-point Likert scale from 1 (not at all) to 4 (very) like before the pandemic, e.g. ‘how important do you consider a vaccination for measles?’ However, measles were replaced by Covid-19. The medical students were also asked about compulsory COVID-19 vaccinations and their reasons for and against this practice; if they refused the COVID-19 vaccination, the students were asked why they did so.

As there was no existing validated questionnaire fitting the study’s purpose, the authors created one by using the think-aloud technique and by conducting a systematic literature research based on the study’s aim. The newly developed questionnaire was previously tested among experts in the field of paediatrics as well as in psychology to determine relevant criteria like objectivity.

### Focus groups

An interview guide was developed to guide the focus group discussions that consisted of open-ended questions intended to evoke the students’ personal attitudes toward vaccinations and vaccination-critical parents, information needed in preparation for a consultation comparable to the one role-played in the teaching and the course’s potential to influence their attitudes. The focus groups were conducted immediately after the teaching session, and each focus group lasted 45– 60 minutes.

### Data processing

All data gathered through the questionnaire and focus groups were pseudonymised in the event that any of the students later wished to withdraw their consent to participate. The focus groups were recorded and transcribed verbatim, and the recordings and transcripts were stored on a secure computer with no internet connection.

### Data analysis

Quantitative data were evaluated using IBM^®^ SPSS^®^ Statistics 27 . 0, and frequencies, percentages, mean values (*M*) and standard deviations (*SD*) were calculated. Data were assessed for normal distribution with the Kolmogorov–Smirnov test, and *T*-tests for independent samples were conducted to compare the responses of the medical students prior to and during the COVID-19 pandemic; the level of significance was set at *p* < 0.05.

Two different reviewers (i.e., CG and TL) employed Mayring [23] qualitative content analysis to assess the focus groups. One reviewer was a paediatrics expert and the other reviewer was an expert in patient–physician communication. Mayring’s seven-step model includes paraphrasing, reduction, summarising for general paraphrases, naming and describing categories, adding examples, building a hierarchy of categories and recoding; the derived categories and associated examples are described in the results. This analysis was conducted using MAXQDA and Microsoft Word.

## Results

A total of 170 (*RR* = 74.8%) medical students completed the quantitative questionnaire, and 59 (*RR* = 65.6%) students participated in the altogether nine focus groups Prior to the pandemic, the average age of the students was *M* = 26.52 (*SD* = 2.51), of whom 75.8% were female. The average age of the medical students during the pandemic was *M* = 25.33 (*SD* = 2.79), and 71.1% were female.

### Students’ attitudes toward vaccinations

#### Quantitative results

All students (100.0%) reported that they had been vaccinated at least once. When asked about the severity of various diseases prior to the pandemic, the medical students ranked measles the most severe (95.4%), followed by mumps (86.3%), rubella (84.8%) and pertussis (80.3%); varicella was rated as the least severe (30.3%). Also, before the pandemic, 97.0% of the students stated that they would have their own children vaccinated against measles, mumps and rubella, 95.5% would have their own children vaccinated against pertussis and 69.7% against varicella. These students strongly disagreed that it was better to experience a disease rather than being vaccinated against it (i.e., measles: 95.5%; mumps: 93.9%; rubella: 92.4%; pertussis: 86.4%); as expected, significantly fewer students (51.5%) felt this way about varicella.

When medical students were asked these same questions during the COVID-19 pandemic, they ranked the measles as being the most severe (94.9%), followed by COVID-19 (82.5%), pertussis (81.5%), mumps (79.4%), rubella (71.1%) and varicella (29.9%). A majority of the students also stated that they would vaccinate their children against measles (95.9%), rubella (93.8%), pertussis (89.7%), COVID-19 (88.8%) and varicella (66.0%). Moreover, they disagreed to a stronger degree that it was better to experience measles, mumps and rubella (100%), pertussis (96.9%) and COVID-19 (89.6%) than to be vaccinated against these illnesses; notably, 90.7% of the peri-pandemic students strongly disagreed that it was better to experience varicella than to be vaccinate against it, which is significantly different from the responses of the pre-pandemic students (*p* < 0.05).

During the COVID-19 pandemic, the medical students viewed vaccinations for several diseases—such as measles, pneumococcal and COVID-19—as important (range: *M* = 3.56, *SD* = 0.54 to *M* = 3.97, *SD* = 0.17). Medical student responses prior to the pandemic yielded similar results as there were no significant differences for the single diseases when compared before and after the _COVID-19 pandemic (range: *M* = 3.26, *SD* = 0.77 to *M* = 3.94, *SD* = 0.24). There were significant differences in the levels of importance students assigned to hepatitis B, HPV and diphtheria before and during the pandemic (diphtheria: *M* = 3.93, *SD* = 0.28 versus *M* = 3.80, *SD* = 0.47, *p* = .032; hepatitis B: *M* = 3.87, *SD* = 0.37 versus *M* = 3.67, *SD* = 0.51, *p* = .004; HPV: *M* = 3.76, *SD* = 0.52 versus *M* = 3.53, *SD* = 0.53, *p* = .006).

Furthermore, we tested if being a parent influenced the results. However, there was no significant effect for being a parent. Saying that, the numbers of students being a parent was quite low with only 7.6%.

#### Vaccination agreement, general attitude and importance

In relation to vaccinations, the medical students were asked about their agreement (or lack thereof), their general attitudes and the perceived importance of various statements on this topic. Most students agreed regarding their attitudes. The pre-pandemic students agreed to a greater extent than the peri-pandemic students that they were interested in having discussions about vaccination and that they would learn more about vaccinations if they ever had children of their own (see Table 1).

**Table 1:** Attitudes toward vaccinations.

	Medical Students Prior to Pandemic (*N* = 66)		Medical Students During Pandemic (*N* = 97)		Statistics
Item	*M*	*SD*	*M*	*SD*	*P*
**What is your general attitude toward vaccinations?**					
I got all recommended vaccinations.	3.94	0.30	3.90	0.31	
I did not have some of the recommended vaccinations, but I do not refuse them.	2.14	1.03	1.94	1.02	
I refuse individual vaccinations.	1.39	0.70	1.28	0.70	
I reject vaccinations in principle.	1.03	0.25	1.00	0.00	
**How much do you agree with the following statements?**					
Vaccinations are important for children’s health.	3.88	0.37	3.87	0.42	
Vaccinations save children.	3.78	0.41	3.80	0.47	
Vaccinations protect against infectious diseases.	3.92	0.27	3.92	0.37	
Vaccinations are harmful and superfluous.	1.08	0.4	1.00	0.00	
I think carefully about each vaccination.	2.3	0.78	2.16	0.84	
Vaccinations primarily serve the interests of pharmaceutical companies.	1.29	0.49	1.22	0.41	
I am not interested in the vaccination discussion.*	1.42	0.64	1.65	0.71	< .039
I will learn more about vaccinations if I ever have children of my own.*	2.97	0.86	2.65	0.97	< .032
**How important or unimportant is it to you that…**					
…you do not infect anyone with diseases that can be vaccinated against?	3.79	0.86	3.76	0.52	
…you help to ensure that diseases for which there are vaccinations no longer occur in the population?	3.85	0.44	3.87	0.34	
…you protect yourself from diseases that can be vaccinated against?	3.86	0.39	3.87	0.34	
…you do not experience any side effects from vaccinations?	2.95	0.64	2.72	0.80	
…others have vaccinated themselves and their children, so you and your family are also protected against contracting diseases?	3.8	0.44	3.63	0.68	

*Note*. All items are rated on a 4–point Likert scale from 1 (not at all) to 4 (very). Items with significant differences are marked with *. Blank cells in the column ‘statistics’ mean p–values > .05.

#### Compulsory vaccinations and reasons for and against vaccinations

**Fig 1:**
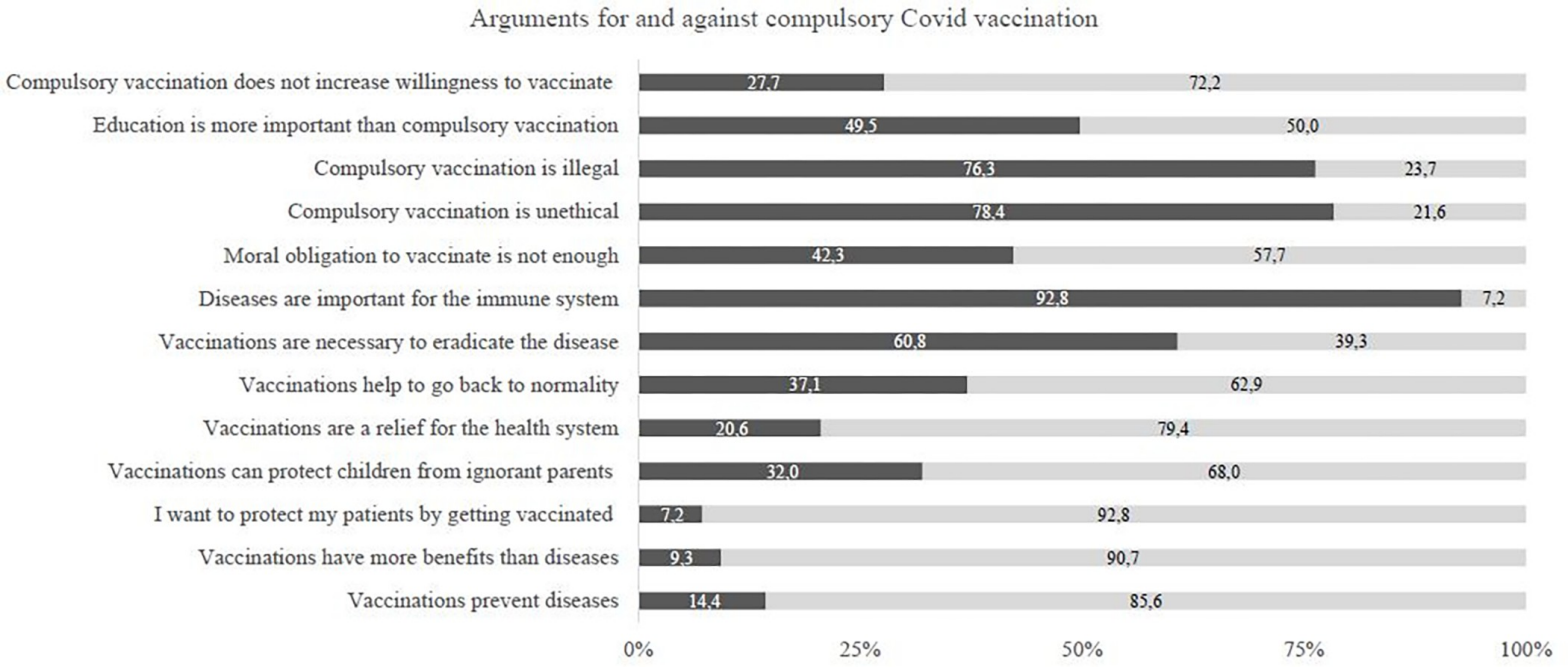
Arguments for and against compulsory Covid–19 vaccination of medical students within in the pandemic.

A significant difference for compulsory vaccination (*p* = .011) was observed between the pre- and peri-pandemic medical students. During the COVID-19 pandemic, the students agreed with this practice to a significantly greater degree (*M* = 3.74; *SD* = 1.18), compared to the student respondents prior to the pandemic (*M* = 3.33; *SD* = 0.66). Moreover, during the pandemic, most of the medical students agreed that compulsory vaccinations should be introduced in specific medical institutions such as hospitals (*M* = 4.09; *SD* = 1.10). The most commonly reported reasons for COVID-19 vaccination were ‘protect patients’ (92.8%), ‘own protection’ (90.7%) and ‘prevention of diseases’ (85.6%) (see Fig 1).

**Fig 2:**
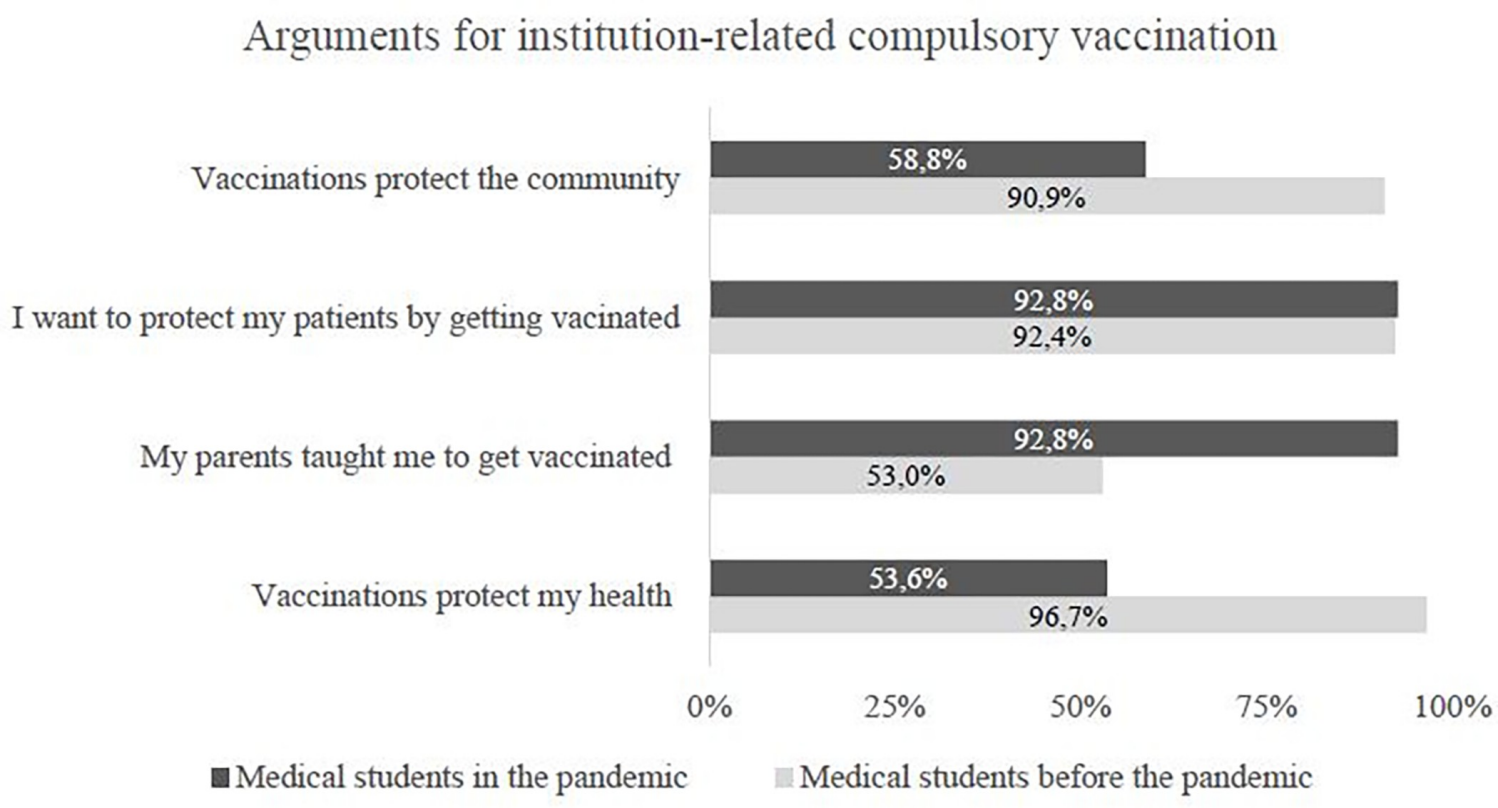
Arguments for institution–related compulsory vaccination in % separated for medical students before and in the pandemic.

When asked to explain their general support for institution-related compulsory vaccination, the peri-pandemic students gave several reasons (see Fig 2).

### Qualitative results of focus groups

Based on the qualitative content analysis of Mayring the following main categories were found: ‘vaccination as general topic’, ‘reasons in favour of compulsory vaccinations’, ‘reasons against compulsory vaccinations’ and ‘patient-physician communication’. Three categories—‘prevention’, ‘relevance’ and ‘reachability’—were derived from the general topic of vaccinations. One student stressed that ‘prevention is important everywhere, not only in paediatrics’ (Student 2). They also saw the advantages to ‘reach children before bad habits or environmental influences become involved’ (Student 8). However, when regarding the relevance of vaccination, they were ambivalent: One student reported that ‘a lot has been achieved by vaccinations, and this success is very demonstrable’ (Student 6). Another student argued that ‘not all vaccinations are necessary for everyone’ (Student 5). They also discussed the controversial topic of compulsory vaccinations and explained why they were in favour or against this practice. Reasons for compulsory vaccination were ‘prerequisite for childcare’, ‘keeping high medical standards’ and ‘possibility of a uniform medical treatment strategy’. Furthermore, they suggested ‘compulsory vaccination with the option that parents can actively object and have the option to refuse a vaccination after consulting with a doctor’ (Student 10). Alternatively, ‘[….] only selected vaccinations’ should be compulsory (Student 15). ‘Curtailment of personal freedom’, ‘needed clarification/education’ and ‘reduced acceptance’ were found as reasons against compulsory vaccination. For example, one student argued that ‘instead of introducing compulsory vaccinations, the offer should be changed and accessibility increased’ (Student 19).

When regarding patient–physician communication, the students focussed on ‘dealing with vaccination-critical parents’ and ‘unvaccinated children’. Both categories were divided into negative and positive handling. Moreover, students strengthened how important it is to inform parents. One student stated that ‘Informational material adapted to parents should be handed out’ (Student 29). Please see Table 2 for more details.

**Table 2:** Categories and examples from medical student focus groups.

**Vaccinations as a General Topic**	
**Category**	**Examples**
Prevention	‘Not only to cure diseases, but to prevent them in the first place.’ (Student 1)
	‘Prevention [through vaccinations] is important everywhere, not only in paediatrics.’ (Student 2)
	‘Society gains a great benefit from vaccinations.’ (Student 3)
	‘The side effects are less [of a problem] than the diseases that can otherwise break out.’ (Student 4)
Relevance (negative)	‘Not all vaccinations are necessary for everyone (e.g., flu, tick-borne encephalitis).’ (Student 5)
Relevance (positive)	‘A lot has been achieved through vaccinations, and this success is very demonstrable.’ (Student 6)
Reachability	‘Especially children can still be reached.’ (Student 7)
	‘You can reach the children before bad habits or environmental influences become involved.’ (Student 8)
**Reasons in Favour of Compulsory Vaccinations**	
Prerequisite for Childcare	‘Without compulsory vaccinations, the child is “at the mercy” of their parents’ decision/worldview.’ (Student 9)
Compulsory vaccination with active contradiction	‘Compulsory vaccination with the option that parents can actively object and have the option to refuse a vaccination after consulting with a doctor.’ (Student 10)
Possibility of a uniform medical treatment strategy	‘Logistical difficulty of treating unvaccinated patients in a doctor’s office.’ (Student 11)
Keeping high medical standards	‘We have to ensure that high standards are maintained.’ (Student 12)
	‘The benefits [of vaccinations] outweigh the risks.’ (Student 14)
Compulsory vaccinations for specific diseases	‘Not for all vaccinations [should be compulsory], only selected vaccinations.’ (Student 15)
**Reasons Against Compulsory Vaccinations**	
Curtailment of personal freedom	‘[Compulsion] makes people feel powerless, especially [when it comes] to their own child.’ (Student 6)
	‘[This would violate the] dignity of the human being.’ (Student 17)
Clarification/education is needed	‘Better education makes more sense than compulsory vaccinations.’ (Student 18)
	‘Instead of introducing compulsory vaccinations, the offer should be changed and accessibility increased.’ (Student 19)
Reduced Acceptance	‘Acceptance of other vaccinations or measures would be reduced as a result.’ (Student 20)
	‘The numbers [of organ donors] would decrease.’ (Student 21)
**Patient–Physician Communication**	
Dealing with vaccination-critical parents (negative handling)	‘Patients should be referred to another doctor.’ (Student 22)
Dealing with vaccination-critical parents (positive handling)	‘They should be educated […] about the benefits and risks of vaccinations, but not referred [to another] doctor’s office.’ (Student 23)
	‘[Doctors] should take [these] people seriously.’ (Student 24)
Dealing with unvaccinated children (negative handling)	‘You should only admit children who are vaccinated to protect other children from infection.’ (Student 25)
Dealing with unvaccinated children (positive handling)	‘Unvaccinated children should not be excluded, but organisational solutions should be found (e.g., a separate waiting room or separate consultation times).’ (Student 26)
	‘Not treating unvaccinated children only shifts the problem and sets up parallel structures.’ (Student 27)
Informing the parents	‘Parents have the right to [ask for] clarification and time should be taken.’ (Student 28)
	‘Informational material adapted to parents should be handed out.’ (Student 29)

#### Evaluation of the teaching concept

The medical students reported that they could benefit from the teaching course. But they wished for more theoretical knowledge on vaccination like having more facts and figures about vaccination. Please see Table 3 for more results.

**Table 3:** Categories and corresponding examples based on medical students’ answers in the focus groups.

Category	Example
Wish for more theoretical knowledge gain	"more facts and figures about vaccination should be communicated". (student 30)
enefit from the course	"the seminar is very useful" (student 31)
	". . .better knowledge of how to conduct a vaccination interview". (student 32)
	"I now feel confident in dealing with vaccine-critical parents.” (student 33)

## Discussion

The present study employed a quantitative questionnaire and focus groups to investigate medical students’ attitudes concerning the importance and relevance of vaccinations and vaccination-critical parents. In this context, a new teaching concept on disease prevention in paediatrics was developed to strengthen the topic of ‘vaccinations’ during medical training; the students reported in the focus groups that they were more confident about dealing with vaccination-critical parents after learning this new teaching concept, and they emphasised the importance of delivering information and officially offering vaccination recommendations to parents. In the quantitative analysis, all students had reportedly been vaccinated at least once, and most agreed it was better to vaccinate against highly infectious diseases such as measles with the potential to be severe; similar responses were given by the pre- and peri-pandemic students. Furthermore, whereas the quantitative data show that a majority of the students were in favour of compulsory vaccinations, the students in the focus groups voiced controversial views on this topic and provided several reasons for the support like maintaining high medical standards or opposition e.g. curtailment of personal freedom to this practice. Moreover, the interviewed students strengthened how important it is to comprehensively inform parents about vaccinations.

### Vaccination relevance

The students agreed that it was important to be vaccinated against highly infectious diseases and that vaccinations could, in fact, prevent these illnesses. They also agreed with the evidence-based rationale for vaccinations: to protect oneself, to protect the community and to eradicate diseases in the population [24, 25]. Vaccination hesitancy seemed to increase as the severity of the disease decreased, however, and the students disagreed as to whether it was better to experience varicella instead of being vaccinated again it, which reveals a lack of knowledge regarding the severe effects of this disease [26, 27]. On the other side, students argued that not all vaccinations were necessary for everyone. Nearly all students (> 90%) agreed that it was very important to vaccinate against measles, mumps and rubella. Varicella, however, were less seen as necessary illness to vaccinate and students argued that it is okay to experience varicella. Furthermore, students reported that a lot has been achieved through vaccinations regarding some illnesses.

Similar to recent studies on medical students’ attitudes toward the COVID-19 vaccination, most of the peri-pandemic students agreed to vaccinate against COVID-19 [28–30].

### Compulsory vaccinations

The quantitative data indicate that most of the surveyed students approved of compulsory vaccinations. Their reported reasons were in line with the evidence-based rationale for vaccination, such as one’s own protection and protecting the community, including patients and society in general [24]; they also considered a physician’s recommendation to be a valid reason for vaccination. This result is in line with the findings of Jungbauer-Gans and Kriwy (31), who concluded that the decision to be vaccinated is dependent on the recommendation of a physician, because these medical professionals are seen as a competent advisors [31].

When asked about compulsory COVID-19 vaccinations, most of the students agreed that they should be introduced in specific medical institutions, such as hospitals. The most commonly reported reasons for COVID-19 vaccination were ‘protect patients’, ‘own protection’ and ‘prevention of diseases’.

Medical students in the focus groups displayed controversial attitudes regarding compulsory vaccination, and the students discussed several reasons why they were in favour of or opposed to compulsory vaccination programmes. Similar to previous studies, the arguments in favour of compulsory vaccination were included the benefits of immunising children, maintaining high medical standards and consistent medical treatment [27, 32, 33]. Notably, in relation to compulsory vaccinations, the fear of reduced vaccination acceptance and the effects of restricted personal freedom were both associated with increased vaccination hesitancy [24, 27].

Several studies have suggested that vaccination hesitancy increases due to misconceptions, missing information and a lack of trust in the healthcare system ; Leask [34] strengthened this argument by demonstrating that poor vaccination educational programmes decreased vaccination readiness. In line with these studies, some students who did not favour compulsory vaccination discussed improving the delivery of information to patients and clarifying information related to being vaccinated.

### Patient–physician communication

Properly informing parents and patients about vaccinations effectively decreases vaccination hesitancy [27, 35]. The interviewed students also insisted that parents should be given sufficient, understandable information on vaccination. According to Gaczkowska & Kirschbaum (2011), 90% of parents named the physician as their primary source of information on vaccinations [36]. Additionally, an investigation of a German governmental health education institution found 79% of the those surveyed reported that they were in favour of vaccinations because their physicians had recommended them [37]. Similarly, 63.3% of the medical students in the present study reported that they trusted their physician. Because trust in a medical expert is perceived as being more important than the actual information they are delivering, establishing a trusting patient–physician relationship is a valuable strategy for decreasing vaccination hesitancy [17, 27, 35].

The interviewed students also discussed ways to deal with vaccination-critical parents. While some of the students suggested that they be refused medical treatment and/or sent to another doctor, most preferred talking about the parents’ fears and misconceptions of vaccinations and recommended that vaccination-critical parents should receive information about the advantages of vaccinations and physicians should provide recommendations [15, 17]. Furthermore, they agreed that rather than exclude unvaccinated children from private practices, physicians should find organisational solutions for these patients, such as separate consultation times or waiting rooms.

### Evaluation of teaching concept

In general, the students assessed the new teaching concept as being useful, and they felt more-prepared to deal with vaccination-critical parents after the course. This indicates that teaching courses on vaccinations should be fully integrated into medical training, because such learning interventions have the potential to improve vaccination acceptance among both physicians and patients [38].

### Limitations

This mixed-method study was limited because the quantitative and qualitative data were obtained from two separate groups of students: one group completed the questionnaire and the other group participated in the focus group; the aim of having separate groups was to avoid student bias. Furthermore, even though we provided the questionnaire to the students during the pandemic, it was too difficult to organise focus groups because of pandemic-related guidelines. In future research, we therefore plan to conduct focus groups on compulsory vaccination with students in different health disciplines.

Moreover, the data represented the students’ self-assessment of their ability to manage vaccine hesitancy but the students’ clinical skills were not tested in this study. In future research, students’ actual clinical skills how to deal with vaccine-critical parents need to be examined (e.g. in an OSCE).

While the students rated the teaching concept as useful and reported that they felt more confident to deal with vaccination-critical parents, this concept could have been evaluated in greater detail. Furthermore, the role-play was not used as a form of assessment of the teaching effectiveness. In future research, the patient–physician communication in a simulated role play should be further investigated to address challenges in delivering relevant information to vaccination-critical parents.

## Conclusion

This mixed-method study investigated medical students’ attitudes toward vaccination relevance, ways to deal with vaccination-critical parents and arguments for and against compulsory vaccinations. While the surveyed students agreed about the importance of vaccinating against highly infectious diseases, their levels of agreement decreased according to the severity of the disease [27]. The interviewed students also considered vaccinations as an important strategy to prevent diseases. However, they reported that not all vaccinations were relevant for everyone. The following conclusions can be derived from the results. Medical students are aware of the advantages as well as the risks of vaccinations and they are able to adequately discuss them. They also benefit from the described vaccination teaching concept when dealing with vaccination-critical parents. Furthermore, because misleading information tends to increase vaccination hesitancy, more vaccination literature should be integrated into medical training as suggested by the medical students in the focus groups [24, 27]. In addition, new teaching initiatives such as the concept presented in the present study should be included in medical training. Strategies learned in this teaching pilot project, such as trustworthy patient–physician communication, delivering understandable information and taking patients’ fears seriously, could also help to change negative attitudes toward COVID-19 vaccinations [7, 9]. It should be noted that the discussion regarding COVID-19 vaccinations may have influenced the students’ attitudes because of the vaccination in general, because of concurrent intensive discussions in which questions of ethical considerations of possible damage caused by the COVID-19 vaccination and the protection of the individual and of society.

## Supporting information

S1 Checklist Standards for Reporting Qualitative Research (SRQR)*. (DOCX)
